# Retinal neurodegeneration in patients with end-stage renal disease assessed by spectral-domain optical coherence tomography

**DOI:** 10.1038/s41598-020-61308-4

**Published:** 2020-03-24

**Authors:** Susanne Jung, Agnes Bosch, Christian Ott, Dennis Kannenkeril, Thomas Dienemann, Joanna M. Harazny, Georg Michelson, Roland E. Schmieder

**Affiliations:** 1 Department of Nephrology and Hypertension, University Hospital Erlangen, Friedrich-Alexander-University Erlangen-Nuremberg (FAU), Erlangen, Germany; 2 Department of Nephrology and Hypertension, Paracelsus Medical School, Nuremberg, Germany; 3 Department of Pathophysiology, University of Warmia and Mazury Olsztyn, Olsztyn, Poland; 4 Department of Ophthalmology, University Hospital Erlangen, Friedrich-Alexander-University Erlangen-Nuremberg (FAU), Erlangen, Germany

**Keywords:** End-stage renal disease, Haemodialysis, Neurodegeneration

## Abstract

Spectral-domain optical coherence tomography (SD-OCT) represents a reliable tool for retinal layer volume and thickness measurement. The aim of this study was to evaluate retinal changes indicating neurodegenerative processes in patients with end-stage renal disease (ESRD) compared to healthy controls. This was a cross-sectional, single-center study comprising 32 ESRD patients and 38 controls. Sectoral retinal nerve fiber layer (RNFL) thickness and retinal layer volumes were obtained by SD-OCT. Age- and gender-adjusted retinal layer volumes such as total retinal volume (p = 0.037), ganglion cell layer volume (GCL, p = 0.003), ganglion cell layer – inner plexiform layer volume (GCL-IPL, p = 0.005) and inner retinal layer volume (IRL, p = 0.042) of the right eye were lower in ESRD patients. Inner plexiform layer volume of both eyes (IPL, right eye: p = 0.017; left eye: 0.044) was reduced, as was RNFL thickness in the temporal superior sector (right eye: p = 0.016). A subgroup analysis excluding patients with diabetes revealed that GCL (p = 0.014) and GCL-IPL volume of the right eye (p = 0.024) and temporal superior sector of the RNFL scan (p = 0.021) in ESRD patients were still significantly thinner. We observed a decrease in several retinal layer volumes and temporal RNFL thickness indicative of retinal neurodegenerative processes in patients with ESRD.

## Introduction

Within the last decade, optical coherence tomography (OCT) of the eye has evolved into a helpful and promising tool to non-invasively evaluate neuronal anatomy and vascular function in patients with various diseases^1–3^. Initially described by Huang *et al*. in 1991^4^, the first *in-vivo* application in a healthy volunteer followed in 1993^5^. Since then, various generations of OCT-devices have been developed. The evolution from time-domain to spectral-domain technology has led to an improvement of image quality, accuracy and velocity^6^.

Nowadays, OCT has been established as a reliable high-resolution imaging technique that is frequently applied in clinical practice for the measurement of retinal layer thickness in patients with age-related macular degeneration^7–9^, glaucoma^10–13^, diabetic retinopathy^14,15^ and other causes of optic neuropathy^16,17^. In addition, the OCT technology has become a valuable diagnostic tool in the assessment of neurodegenerative disorders, allowing the quantification of subtle changes in retinal nerve fiber layer (RNFL) thickness in patients with multiple sclerosis or Parkinson’s disease^18–22^. A decrease in RNFL thickness or volume evaluated by OCT thereby represents an indicator of atrophy due to axonal and neuronal loss and multiple studies have shown that these alterations can be detected even earlier than through funduscopy^17,23^. Therefore, OCT allows a reliable estimation of axonal integrity of the anterior visual pathway^3,23,24^.

In patients with ESRD, alterations like lenticular opacities, changes in choroidal and central corneal thickness, retinal layer thickness as well as intraocular pressure can be observed due to major fluctuations in fluid balance leading to changes in systemic and ocular hemodynamics^25^. Additionally, a significant thinning of the RNFL in patients with end-stage renal disease (ESRD) has been reported^26–28^. These alterations determined by OCT examination may be indicative of neurodegenerative mechanisms affecting the visual function in those patients and need to be further explored.

To our knowledge, long-term alterations of both RNFL thickness and volumes of each retinal layer comprising a more complete analysis of neurodegenerative changes have not yet been investigated in patients with ESRD. Therefore, the aim of the present study is to evaluate neuronal retinal alterations in patients with ESRD in comparison to healthy controls not only by means of circular scans of the RNFL but by additional evaluation of each retinal layer volume, especially the layers containing parts of the ganglion cells determined by spectral domain (SD) OCT.

## Results

### Study population

Baseline characteristics of the study population (n = 70) are shown in Table 1. The group of patients with ESRD included 11 female (34%) and 21 male (65%) patients aged 60.3 ± 15 years with an average office BP of 130.7 ± 25/74.0 ± 11 mmHg and a mean dialysis duration of 97.5 ± 110.2 months. The group of healthy controls included 28 female (74%) and 10 male (26%) subjects aged 57.9 ± 10 years with an average office BP of 128.1 ± 13/77.3 ± 8.8 mmHg and a mean serum creatinine level of 0.8 ± 0.1 mg/dl. Comparing the two groups, there were no significant differences in office systolic and diastolic or mean BP as well as body mass index (BMI), HbA1c and FPG. Patients undergoing HD showed significant differences in total cholesterol, low density lipoprotein (LDL, both p < 0.001) and high density lipoprotein (HDL, p = 0.018) as well as hemoglobin (p < 0.001) and hematocrit (p = 0.001) in comparison to healthy controls.

**Table 1:** Clinical characteristics of the study population.

	Healthy controls (n = 38)	HD (n = 32)	p-value
Age [years]	57.9 ± 10	60.3 ± 15	0.468
Male gender	10 (26%)	21 (66%)	**0.001**
Duration of dialysis [months]	—	97.5 ± 110.2	—
Systolic office BP [mmHg]	128.1 ± 13	130.7 ± 25	0.594
Diastolic office BP [mmHg]	77.3 ± 8.8	74.0 ± 11	0.079
Mean arterial pressure [mmHg]	95.5 ± 8.4	94.3 ± 15	0.681
BMI [kg/m^2^]	24.9 ± 3.3	24.8 ± 3.6	0.875
HbA1c [%]	5.5 ± 0.3	5.7 ± 1.0	0.735
FPG [mg/dl]	90.2 ± 10	95.8 ± 32	0.454
Creatinine [mg/dl]	0.8 ± 0.1	9.8 ± 2.7	**<0.001**
Total cholesterol [mg/dl]	232.3 ± 40	188.5 ± 46	**<0.001**
LDL cholesterol [mg/dl]	155.9 ± 32	119.6 ± 36	**<0.001**
HDL cholesterol [mg/dl]	61.7 ± 12	48.5 ± 15	**0.018**
Hemoglobin [g/dl]	13.9 ± 1.2	11.8 ± 1.5	**<0.001**
Hematocrit [%]	41.1 ± 3.4	36.1 ± 5.6	**0.001**

Data are given as mean ± SD. HD – hemodialysis, BP – blood pressure, BMI – body mass index, HbA1c – glycated hemoglobin, FPG – fasting plasma glucose, eGFR – estimated glomerular filtration rate, LDL – low-density lipoprotein, HDL – high-density lipoprotein.

### SD-OCT measurements

**Figure 1:**
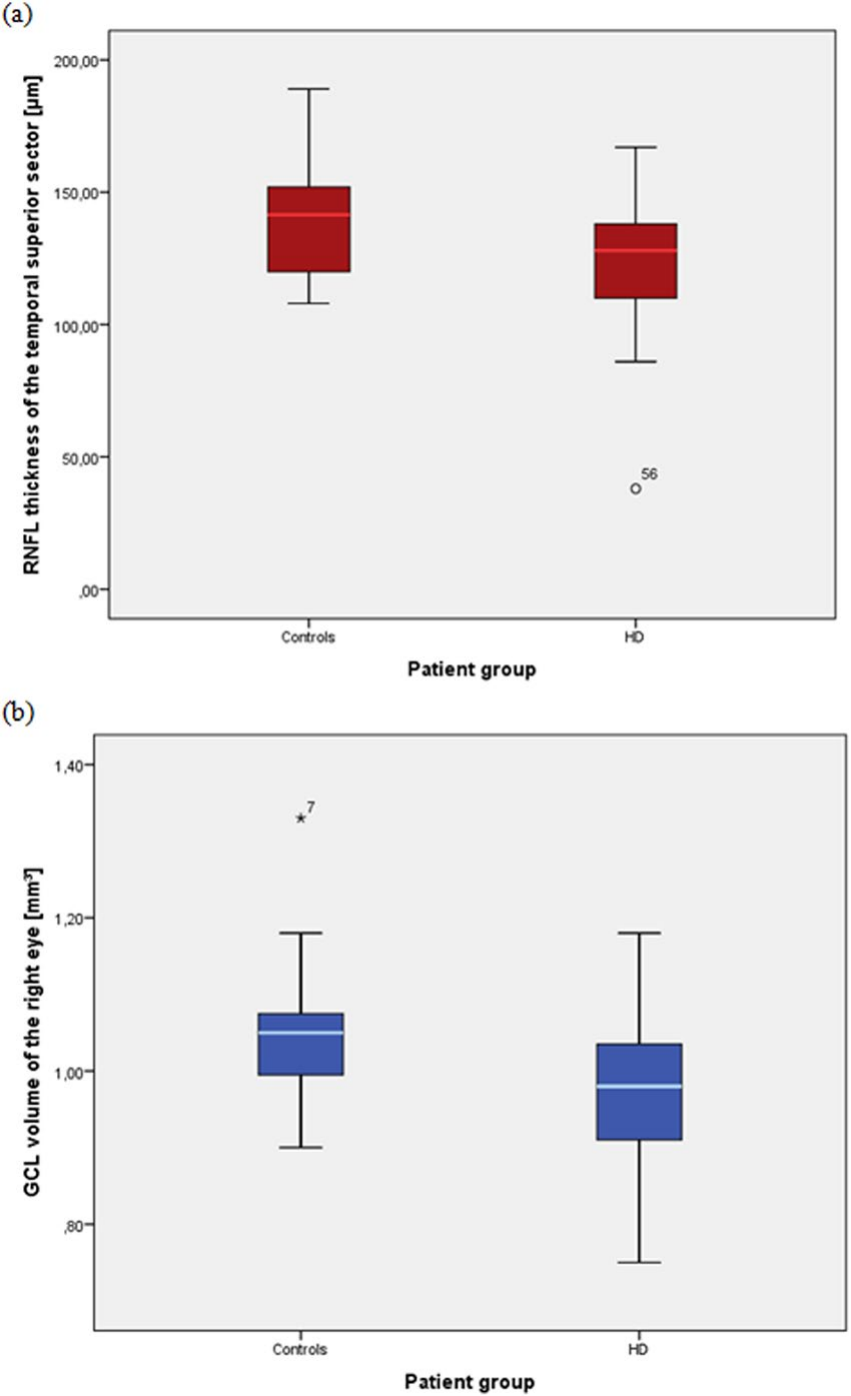
Significant differences in RNFL thickness and GCL volume between the two groups. Pathological thinning of the temporal superior sector of the circular RNFL scan (**a**) and the GCL volume of the right eye (**b**) in patients undergoing HD compared to controls; HD - hemodialysis, RNFL – retinal nerve fiber layer, GCL – ganglion cell layer.

As to retinal layer volume measurement, age-adjusted total retinal volume of both eyes (right eye: p = 0.014; left eye: p = 0.046) as well as GCL volume (right eye: p = 0.001; left eye: p = 0.027, see Fig. 1), IPL volume (right eye: p = 0.004; left eye: p = 0.011), GCL-IPL volumes (right eye: p = 0.001; left eye: p = 0.018) of both eyes and IRL volume of the right eye (p = 0.015) were significantly lower in patients with ESRD than in healthy subjects. After additional gender-adjustment, total retinal volume (p = 0.037), GCL (p = 0.003), GCL-IPL (p = 0.005) and IRL volumes (p = 0.042) of the right eye as well as IPL of both eyes (right eye: p = 0.017; left eye: p = 0.044) were still significantly lower in patients with ESRD.

RNFL thickness and retinal layer volume measurements obtained by SD-OCT are presented in Tables 2 and 3. With respect to RNFL thickness, age-adjusted temporal superior sectors of the circular RNFL scan of both eyes was significantly thinner in patients undergoing HD compared to healthy controls (right eye: p = 0.008; left eye: p = 0.018). After additional adjustment for gender, the significant difference in thickness of the temporal superior sector of the right eye (p = 0.016) was persistent (Fig. 1).

**Table 2:** Sectorial RNFL thickness analysis in CKD patients on HD versus healthy controls obtained by SD-OCT.

Circular scans [µm]	Healthy controls (n = 38)	HD (n = 32)	p-value	p-value (age-adjusted)	p-value (age- and gender-adjusted)
Global (OD)	97.32 ± 9.36	94.38 ± 21.74	0.536	0.479	0.653
Global (OS)	96.62 ± 10.81	94.39 ± 15.78	0.534	0.696	0.902
Temp. (OD)	78.08 ± 15.32	75.46 ± 24.82	0.608	0.531	0.553
Temp. (OS)	66.49 ± 13.22	68.54 ± 27.10	0.723	0.585	0.389
Temp. sup. (OD)	138.53 ± 20.35	122.58 ± 26.73	**0.010**	**0.008**	**0.016**
Temp. sup. (OS)	134.05 ± 19.97	117.92 ± 30.16	**0.022**	**0.018**	0.175
Temp. inf. (OD)	144.13 ± 22.07	138.42 ± 34.12	0.426	0.423	0.747
Temp. inf. (OS)	132.62 ± 25.97	123.27 ± 25.52	0.162	0.190	0.510
Nasal (OD)	68.16 ± 14.56	69.42 ± 28.50	0.819	0.748	0.927
Nasal (OS)	73.68 ± 14.23	78.89 ± 18.55	0.212	0.146	0.329
Nasal sup. (OD)	99.95 ± 19.06	100.25 ± 32.54	0.963	0.827	0.855
Nasal sup. (OS)	112.62 ± 19.49	111.15 ± 31.97	0.836	0.963	0.942
Nasal inf. (OD)	103.45 ± 20.66	102.96 ± 38.74	0.955	0.944	0.792
Nasal inf. (OS)	113.27 ± 21.85	107.92 ± 25.62	0.377	0.509	0.178

Data are given as mean ± SD. HD – hemodialysis, temp – temporal, sup – superior, inf – inferior, OD – oculus dexter, OS – oculus sinister

**Table 3:** Retinal layer volume analysis in CKD patients on HD versus healthy controls obtained by SD-OCT.

Retinal layer volumes [mm³]	Healthy controls (n = 38)	HD (n = 32)	p-value	p-value (age-adjusted)	p-value (age- and gender-adjusted)
Retina (OD)	8.51 ± 0.41	8.22 ± 0.45	**0.010**	**0.014**	**0.037**
Retina (OS)	8.50 ± 0.40	8.26 ± 0.48	**0.034**	**0.046**	0.074
GCL (OD)	1.05 ± 0.08	0.96 ± 0.10	**<0.001**	**0.001**	**0.003**
GCL (OS)	1.04 ± 0.10	0.98 ± 0.12	**0.016**	**0.027**	0.119
IPL (OD)	0.88 ± 0.07	0.83 ± 0.08	**0.003**	**0.004**	**0.017**
IPL (OS)	0.88 ± 0.07	0.82 ± 0.07	**0.007**	**0.011**	**0.044**
INL (OD)	0.95 ± 0.08	0.95 ± 0.10	0.894	0.951	0.498
INL (OS)	0.96 ± 0.08	0.94 ± 0.10	0.532	0.606	0.157
OPL (OD)	0.82 ± 0.06	0.80 ± 0.07	0.222	0.233	0.552
OPL (OS)	0.81 ± 0.05	0.80 ± 0.06	0.446	0.438	0.543
ONL (OD)	1.67 ± 0.20	1.59 ± 0.22	0.158	0.202	0.235
ONL (OS)	1.69 ± 0.18	1.62 ± 0.22	0.156	0.195	0.158
PR (OD)	2.24 ± 0.08	2.22 ± 0.10	0.414	0.426	0.423
PR (OS)	2.26 ± 0.07	2.23 ± 0.09	0.216	0.217	0.146
RPE (OD)	0.39 ± 0.04	0.39 ± 0.07	0.601	0.668	0.678
RPE (OS)	0.40 ± 0.04	0.40 ± 0.06	0.779	0.869	0.894
IRL (OD)	6.27 ± 0.38	6.00 ± 0.43	**0.011**	**0.015**	**0.042**
IRL (OS)	6.24 ± 0.38	6.02 ± 0.50	0.056	0.076	0.126
RNFL (OD)	0.9 ± 0.08	0.98 ± 0.12	0.366	0.344	0.997
RNFL (OS)	0.9 ± 0.12	0.89 ± 0.10	0.949	0.809	0.650
GCL-IPL (OD)	1.93 ± 0.15	1.79 ± 0.67	**0.001**	**0.001**	**0.005**
GCL-IPL (OS)	1.92 ± 0.17	1.80 ± 0.20	**0.012**	**0.018**	0.076

Data are given as mean ± SD. HD– hemodialysis, RNFL – retinal nerve fiber layer, GCL – ganglion cell layer, IPL – inner plexiform layer, INL – inner nuclear layer, OPL – outer plexiform layer, ONL – outer nuclear layer, RPE– retinal pigment epithelium, IRL – inner retinal layer, RNFL – retinal nerve fiber layer, PR – photo receptor layer, OD – oculus dexter, OS –oculus sinister.

To eliminate potential confounding effects of diabetes mellitus on RNFL thickness and retinal layer volumes in our patients, we performed a subgroup analysis excluding the 7 patients with type 2 diabetes mellitus (T2DM) on chronic maintenance HD. This subgroup comprised 25 subjects (9 female and 16 male individuals), that were compared to healthy controls.

The results of this subgroup analysis are presented in Table 4. With respect to retinal layer volumes in this subgroup analysis, age-adjusted GCL volume of the right eye (p = 0.005), IPL volume of both eyes (right eye: p = 0.021; left eye: p = 0.025) and GCL-IPL volume of both eyes (right eye: p = 0.009; left eye: p = 0.042) were significantly thinner in HD patients compared to healthy controls. Age- and gender-adjusted analysis revealed a significant difference in GCL volume of the right eye (p = 0.014), GCL-IPL volume of the right eye (p = 0.024) as well as INL volume of the left eye in this subgroup (p = 0.015).

**Table 4:** Circular sectoral papillary RNFL thickness scans and retinal layer volumes in CKD patients on HD without T2DM versus healthy controls.

Circular scans [µm]	Healthy controls (n = 38)	HD without T2DM (n = 25)	p-value	p-value (age-adjusted)	p-value (age- and gender-adjusted)
Temp. sup. right	138.53 ± 20.35	125.00 ± 20.09	**0.021**	**0.023**	**0.021**
Temp. sup. (OS)	134.05 ± 19.97	123.10 ± 29.02	0.099	0.136	0.519
**Retinal layer volumes [mm³]**	**Healthy controls (n** = **38)**	**HD without T2DM (n** = **25)**	**p-value**	**p-value (age-adjusted)**	**p-value (age- and gender-adjusted)**
Retina (OD)	8.51 ± 0.41	8.27 ± 0.49	0.055	0.083	0.161
Retina (OS)	8.50 ± 0.40	8.26 ± 0.48	**0.047**	0.077	0.143
GCL (OD)	1.05 ± 0.08	0.97 ± 0.11	**0.003**	**0.005**	**0.014**
GCL (OS)	1.04 ± 0.10	0.98 ± 0.12	**0.036**	0.067	0.235
IPL (OD)	0.88 ± 0.07	0.83 ± 0.79	**0.014**	**0.021**	0.057
IPL (OS)	0.88 ± 0.07	0.82 ± 0.92	**0.013**	**0.025**	0.076
INL (OD)	0.95 ± 0.08	0.93 ± 0.07	0.279	0.364	0.161
INL (OS)	0.96 ± 0.08	0.92 ± 0.65	0.073	0.084	**0.015**
IRL (OD)	6.27 ± 0.38	6.03 ± 0.48	**0.040**	0.061	0.132
IRL (OS)	6.24 ± 0.38	6.01 ± 0.48	0.052	0.085	0.170
GCL-IPL (OD)	1.93 ± 0.15	1.80 ± 0.19	**0.005**	**0.009**	**0.024**
GCL-IPL (OS)	1.92 ± 0.17	1.80 ± 0.21	**0.022**	**0.042**	0.145

Data are given as mean ± SD. HD– hemodialysis, T2DM – type 2 diabetes mellitus, temp. – temporal, sup. – superior, GCL – ganglion cell layer, IPL – inner plexiform layer, INL – inner nuclear layer, IRL – inner retinal layer, OD – oculus dexter, OS – oculus sinister.

Similarly to the whole group of patients with ESRD, the age-adjusted temporal superior sector of the circular RNFL scan of the right eye was significantly thinner than in the control group (p = 0.023). This difference persisted after additional adjustment for gender (p = 0.021).

There was no significant correlation between above mentioned layer thickness and volume measurements and duration of dialysis in the group of patients with ESRD.

## Discussion

In the present study, we analyzed and compared changes in RNFL thickness and retinal layer volumes, especially the layers containing parts of the ganglion cells in two different groups of subjects, patients with ESRD and healthy controls. This is the first study to evaluate alterations of RNFL thickness as well as volumes of each retinal layer of both eyes separately in patients with ESRD. This approach reflects a more complete analysis of retinal neurodegenerative alterations than just assessing one parameter (e.g. RNFL thickness only) or taking both eyes together in one analysis, as done in previous studies^26,28,29^. In comparison to healthy controls, we found a significant decrease in several retinal layer volumes and retinal nerve fiber decrease as indicated by a thickness reduction of the temporal superior sector of the circular RNFL scan in patients with ESRD. Thus, our findings suggest neurodegenerative alterations in ganglion cell volumes and RNFL thickness in those patients.

In the literature, there are only few studies describing the impact of ESRD or HD on retinal thickness parameters. None of them examined retinal layer volumes in addition to RNFL thickness. Demir *et al*. compared 66 eyes of 33 patients with ESRD undergoing HD or peritoneal dialysis to 20 healthy controls and reported a significant thinning of the inferior and temporal quadrants of RNFL in patients undergoing HD, measured by OCT-3. In this study, the patients undergoing HD were on average younger (41.8 ± 10.8 years) than our study subjects (60.3 ± 15 years). Furthermore, duration of dialysis treatment was significantly shorter (42.05 ± 29.96 months) compared to our patients (97.5 ± 110.2 months)^28^. Atilgan *et al*. reported a significant thinning of the RNFL in 40 eyes of 20 patients receiving HD compared to 68 eyes of 34 healthy controls. The average age of HD patients was 37.7 ± 12.2 years and 35.5 ± 13.97 years in the control group and the duration of dialysis was significantly shorter (51.5 ± 36.96 months) than in our study subjects^29^. Pahor *et al*. examined 24 eyes of 12 HD patients and compared them with 32 eyes of 16 healthy controls. OCT of both eyes thereby revealed a significant thinning of total retina in all quadrants in the group of patients with ESRD. With an average age of 50.0 ± 11,1 years in the group of HD patients and 47,3 ± 7,3 years in the control group, those patients were younger than in the present study, but the duration of dialysis treatment (100.8 ± 42 months) was similar to our patients^26^. In contrast to the above mentioned studies in which left and right eyes were analyzed together, we analyzed each eye separately and differences were more prominent in the right than in the left eye, suggesting to initially focus on the right eye to detect early signs of neurodegenerative processes. Differences between right and left side have been previously described for other parameters, e.g. carotid intima-media thickness^30^. To learn more about the potential difference between right and left eye, more studies evaluating both eyes separately would be of major research interest. Furthermore, in daily clinical practice, both eyes are evaluated separately as well. Therefore, this diagnostic approach offers a more accurate, clinically valid analysis and should be applied more frequently in future studies.

In the group of patients with ESRD, we found no significant correlation between retinal layer thickness or volume on the one hand and duration of dialysis on the other hand. These findings are in accordance with the above mentioned study by Pahor *et al*., in which no significant correlation between retinal thickness and duration of dialysis was observed^26^. These findings might be due to the small sample size of the groups.

In all these previous studies, patients with T2DM have been excluded previously. To eliminate potentially interfering effects of T2DM and HD or ESRD on RNFL thickness and retinal layer volume, we performed a subgroup analysis excluding all patients with ESRD suffering from T2DM. In a previous study, we observed that T2DM affected retinal layer volumes in patients without renal disease suffering from T2DM (Jung *et al*., “Neuronal versus vascular retinal changes in patients with type 2 diabetes mellitus compared to healthy controls”, yet unpublished data from our group). In this exploratory subanalysis, we detected significant differences in RNFL thickness of the temporal superior sector and several retinal layer volumes.

This distinction is of importance to verify the isolated effect of homeostatic disorders due to ESRD and HD on retinal layer volume, as retinal alterations, as a sign of neuronal degeneration have been previously described in patients with T2DM^31–33^. For example, a cross-sectional study by Li *et al*. evaluating differences in retinal layer thickness by means of OCT showed a significant thinning of the layers containing parts of the ganglion cells in patients with T2DM even in the absence of early signs of diabetic retinopathy^32^. In 25 subjects with minimal diabetic retinopathy, van Dijk *et al*. found significant decreases in RNFL, IPL and ganglion cell thickness indicating neurodegenerative processes in the course of disease^31^. Thus, we found various retinal layer volume and RNFL thickness alterations even in the subgroup without T2DM, indicating an isolated effect of ESRD and HD on retinal neuronal alterations.

Another cardiovascular risk factor which might represent a confounding factor is the lipid profile. The influence of lipid levels on retinal layer thickness has been previously investigated. A subgroup analysis of the Gutenberg Health Study (GHS) examining the influence of various factors on retinal nerve fiber layer (RNFL) thickness in a total of 1973 subjects showed no significant association between RNFL thickness and lipid levels in the multivariable model^34^. Similarly, the MIPH Eye&Health Study - investigating potential determinants of peripapillary RNFL thickness - found no association between the latter and lipid levels^35^. The same results were found in a population-based sample of 542 subjects from the Singapore Chinese Eye Study^36^.

Optic neuropathy represents a common complication in patients treated with HD due to renal failure^37^. In the literature, various contributing factors for optic neuropathy in patients with ESRD have been postulated. Knox *et al*. reported a neurotoxic form of optic neuropathy in uremic patients^38^. Additionally, comorbidities frequently observed in HD patients such as hypertension, atherosclerosis and glucose intolerance can lead to ischemic optic neuropathy as described by Haider *et al*.^37^. The above mentioned findings of retinal thinning in patients with ESRD by Pahor *et al*. were attributed to subclinical chronic ischemic retinopathy caused by obstructive carotid artery disease^26^.

Early detection of neurodegenerative changes not only plays an important role in the treatment of ophthalmological and neurological diseases such as for example glaucoma or multiple sclerosis, but also in internal medicine. Just like subjects with diabetes mellitus or hypertension regularly undergo ocular examination, subjects suffering from ESRD might undergo ocular examination as well in the future. Therefore, SD-OCT could possibly become part of routine diagnostic screening to prevent early end-organ damage in clinical practice.

Our findings support the occurrence of retinal neurodegeneration and optic atrophy in patients with ESRD. The question remains whether the observed changes are caused by solely ocular or also cerebral, trans-synaptic neurodegenerative processes between retinal ganglion cells (the 3^rd^ neuron of the visual pathway) and lateral geniculate nucleus (nucleus of the 4^th^ neuron) as well as visual cortex. To quantify axonal damage and further investigate the structural integrity of white matter structures, 3T-magnetic resonance imaging (MRI)-based technique of diffusion tensor imaging (DTI) would be a non-invasive well-established diagnostic tool^39–41^. This method, based on the random motion of water molecules associated with their thermal energy at body temperature, has already been applied successfully to characterize trans-synaptic degenerative processes in glaucoma patients^42–44^.

Our study is not without limitations. First, this is a cross-sectional study with a small sample size for each group, which did not allow for identification of pathogenetic mechanisms of our findings. Second, SD-OCT measurements were only done once for each participant at the same time of the day 1–2 hours after hemodialysis. To eliminate the acute effects of the hemodialysis session, it would have seemed advisable to perform two OCT-measurements, namely shortly before and after HD. However, previous studies did not find any significant acute influence of HD on OCT measurements^28,45,46^. Third, age- and gender-adjustment made some of our findings less prominent than only age-adjusted analysis, suggesting that gender modifies the extent of neurodegenerative alterations. However, our study was too small to separately analyze male and female subjects and derivate solid conclusions.

In conclusion, we found several differences in GCL volumes and temporal RNFL thickness, indicating severe neurodegenerative retinal alterations in patients with ESRD in comparison to healthy controls. The evaluation of both, each retinal layer volume separately and sectoral RNFL thickness allowed us to precisely analyze these changes. Furthermore, SD-OCT represents a valuable, accurate and sensitive high-resolution imaging technique to explore early retinal neurodegenerative processes in patients with ESRD. Further longitudinal studies in larger cohorts of patients are needed to assess the pathogenetic mechanisms of alterations in retinal layer thickness and volume during follow-up.

## Methods

### Study design

This is a cross-sectional, observational single center study comprising patients with ESRD and healthy individuals. Patients were examined at the Clinical Research Center of the Department of Nephrology and Hypertension, University of Erlangen-Nuremberg, Germany (www.crc-erlangen.de). Participants with ESRD were recruited directly from our dialysis unit and healthy controls by means of advertisements in local newspapers. Patients who were eligible and gave their written informed consent were enrolled consecutively. Written informed consent was obtained from each subject before study inclusion. The studies were conducted according to the tenets of the Declaration of Helsinki and the principles of good clinical practice guidelines. The study protocol has been approved by the local Ethics Committee of the University of Erlangen-Nuremberg.

### Study population

A total of 70 consecutive subjects were included into the study. The group of healthy controls consisted of 38 individuals aged 40 years and older without any relevant pre-existing medical condition or medication which might possibly influence OCT measurement. Subjects with pre-existing glaucoma, macular degeneration, diabetic retinopathy or uncontrolled hypertension and patients who had previously undergone laser coagulation were excluded. Glaucoma, macular degeneration and diabetic retinopathy were defined according to the latest respective guidelines (Guidelines of the Professional Association of German Ophthalmologists eV and the German Ophthalmology Society, Guidelines No. 15, 20 and 21). Subjects were excluded in case of presence of one of the diseases in their medical records or in case of any signs of the diseases in a funduscopic evaluation which took place prior to study inclusion. The group of patients with ESRD included 32 subjects out of 95 subjects undergoing chronic maintenance HD in our dialysis center in Nuremberg.

### Clinical parameters

Demographic data of all participants were obtained and a fasting blood sample was taken to measure fasting plasma glucose (FPG), glycated hemoglobin (HbA1c), serum creatinine, lipid levels and other biochemical parameters. Assessment of office blood pressure (BP) was carried out in standard fashion by validated devices (DINAMAP® PRO 100V2, GE Critikon) in a seated position after 5 minutes of rest according to ESH/ESC guideline recommendations^47^.

### Ocular examination

All study subjects underwent ocular examination by the same experienced group of trained investigators in a dark room without pupil dilation. In the group of patients with ESRD, OCT measurements were done after dialysis treatment. After correct positioning of the patient, retinal layer measurements of both eyes were obtained using a high-speed, high-resolution SD-OCT system (Spectralis® OCT, Heidelberg Engineering, Heidelberg, Germany). For this purpose, a super luminescence diode (SLD) creates an infrared beam with an average wavelength of 870 nm. In order to prevent motion artefacts, SD-OCT contains an eye tracking system to compensate minimal eye movements during examination.

**Figure 2:**
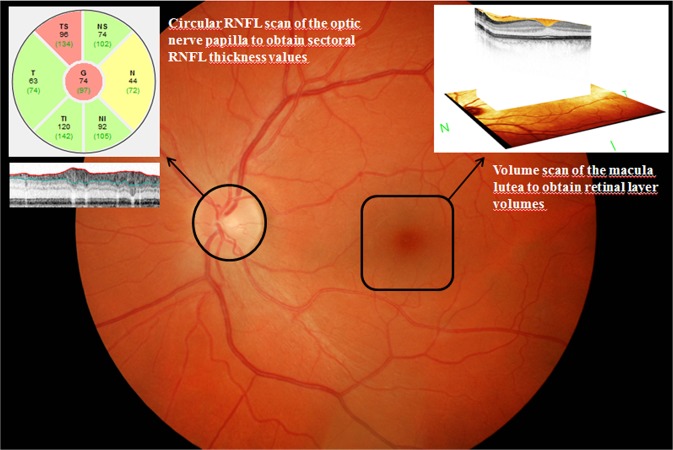
Sectoral RNFL thickness evaluation by means of a circular peripapillary RNFL scan and retinal volume analysis by means of a macular volume scan. RNFL – retinal nerve fiber layer, NS – nasal superior, NI – nasal inferior, N – nasal, TS – temporal superior, TI – temporal inferior, T – temporal, G – global.

**Figure 3:**
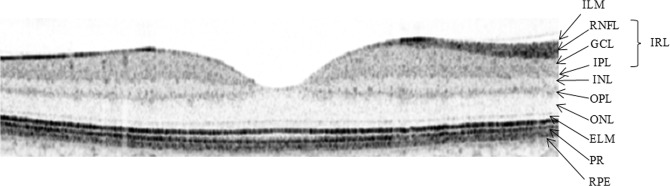
Retinal layer anatomy. ILM – internal limiting membrane, RNFL – retinal nerve fiber layer, GCL-IPL – ganglion cell layer-inner plexiform layer, IRL – inner retinal layer, INL – inner nuclear layer, OPL – outer plexiform layer, ONL – outer nuclear layer, ELM – external limiting membrane, PR – photoreceptor layer, RPE – retinal pigment epithelium.

**Figure 4:**
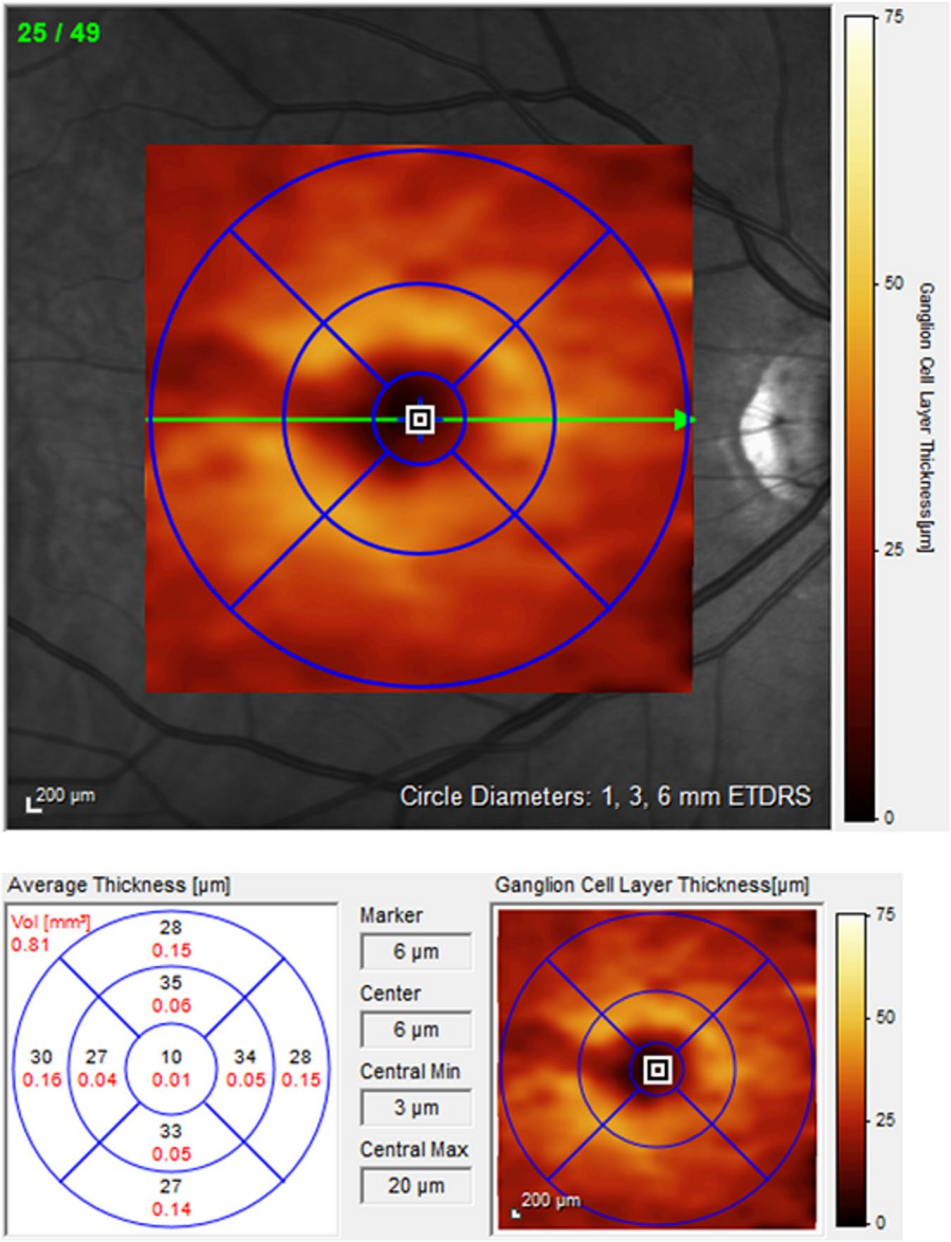
GCL volume scan of a patient with ESRD. Pathological thinning of the GCL; ESRD – end-stage renal disease. GCL – ganglion cell layer, VOL – volume, Min – minimal, Max – maximum, ETDRS – early treatment diabetic retinopathy study.

Macular retinal layer volumes of both eyes were determined by means of computerized segmentation, comprising total retinal volume but also each layer separately from outside to inside (see Figs. 2–4), beginning with RNFL, ganglion cell layer (GCL), inner plexiform layer (IPL), inner nuclear layer (INL), outer plexiform layer (OPL), outer nuclear layer (ONL) up to the photoreceptor layer (PRL) and retinal pigment epithelium (RPE). Additionally, GCL-IPL volume was calculated as the sum of GCL and IPL volume. Similarly, inner retinal layer (IRL) volume was calculated as the sum of RNFL, GCL and IPL volume, reflecting all three layers containing parts of the ganglion cells – axons in the RNFL, cell bodies in the GCL and dendrites in the IPL.

**Figure 5:**
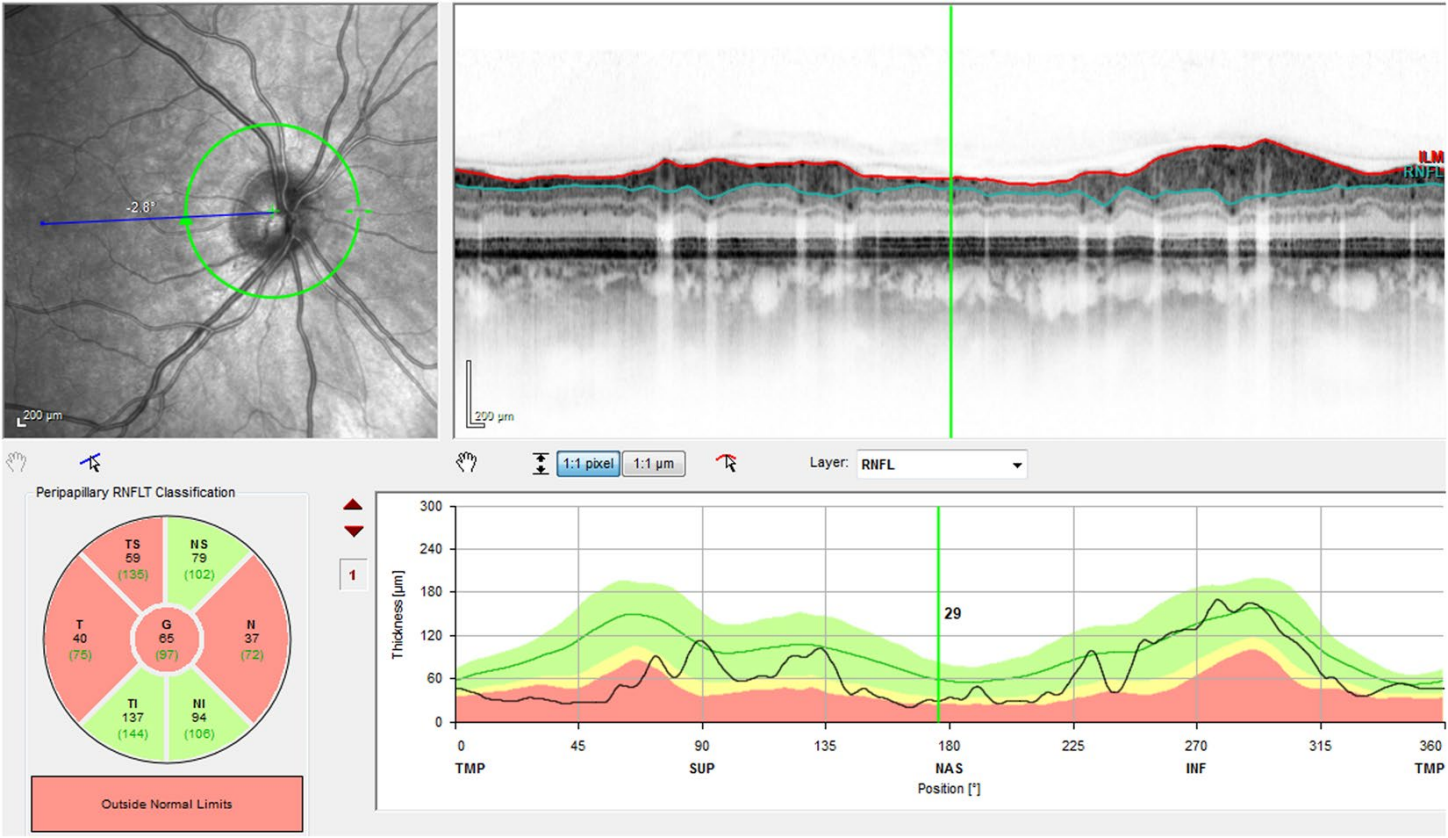
Peripapillary circular RNFL scan of a patient with ESRD. Pathological thinning of several RNFL sectors. ESRD – end-stage renal disease, RNFL – retinal nerve fiber layer, NS – nasal superior, NI – nasal inferior, N – nasal, TS – temporal superior, TI – temporal inferior, T – temporal, G – global, TMP – temporal, NAS – nasal, SUP – superior, INF – inferior.

By means of a circular scan (average diameter 3.45 mm) which had to be positioned correctly around the participant’s pupil, SD-OCT measurements of 1024 points in the optic disc area were performed to determine RNFL thickness in six different sectors (nasal, nasal superior and inferior, temporal, temporal superior and inferior, Figs. 2 and 5). The resulting images were then analyzed and compared digitally to a normative database of n = 861 subjects by means of the Heidelberg Eye Explorer software (HEYEX) and subsequently charted as line and circle diagrams in three different colours (green: within the normal range; yellow: marginal; red: beyond the normal range).

Ocular examination was performed by the same experienced and specially trained investigators who were blinded to the respective group of subjects to reduce bias.

### Statistical analysis

All analyses were performed using SPSS software, version 21.0 (IBM Corporation, Chicago, IL, USA). Data are expressed as mean ± standard deviation (SD). Normal distribution of data was confirmed by Kolmogorov-Smirnov test before further analysis. Normally distributed data were compared by unpaired Student’s t-test. Not normally distributed data were compared using Wilcoxon rank sum test for non-parametric variables. A two-sided p-value < 0.05 was considered statistically significant. P-values were adjusted for age and gender. Bivariate correlation analyses were performed using Pearson’s test for normally distributed parameters and Spearman’s test for not normally distributed data.

## Data Availability

The datasets generated during and/or analysed during the current study are available from the corresponding author on reasonable request.
